# Enhancing the Efficacy of HIPEC Through Bromelain: A Preclinical Investigation in Appendiceal Cancer

**DOI:** 10.1245/s10434-024-15355-0

**Published:** 2024-05-04

**Authors:** Nadeem Wajih, Richard A. Erali, Steven D. Forsythe, Cecilia R. Schaaf, Perry Shen, Edward A. Levine, Shay Soker, David L. Morris, Konstantinos I. Votanopoulos

**Affiliations:** 1 Wake Forest Department of General Surgery, Wake Forest Organoid Research Center (WFORCE), Wake Forest University School of Medicine, Winston-Salem, NC USA; 2 Wake Forest Institute of Regenerative Medicine, Wake Forest University School of Medicine, Winston-Salem, NC USA; 3 Wake Forest Comprehensive Cancer Center, Wake Forest University School of Medicine, Winston-Salem, NC USA; 4 Department of Cancer Biology, Wake Forest University School of Medicine, Winston-Salem, NC USA; 5 Department of Comparative Medicine, Wake Forest University School of Medicine, Winston-Salem, NC USA; 6 Department of Surgery, St. George Hospital, University of New South Wales, Sydney, Australia; 7 Section of Surgical Oncology, Department of Surgery, Wake Forest University School of Medicine, Winston-Salem, NC USA

**Keywords:** Appendiceal cancer, Organoids, Bromelain, HIPEC

## Abstract

**Introduction:**

Appendiceal cancer (AC) excessive mucin production is a barrier to heated intraperitoneal chemotherapy (HIPEC) drug delivery. Bromelain is a pineapple stem extract with mucolytic properties. We explored bromelain treatment effects against mucinous AC in a patient-derived tumor organoid (PTO) model and an AC cell line.

**Patients and Methods:**

PTOs were fabricated from tumor specimens obtained from patients with AC undergoing cytoreductive surgery with HIPEC. PTOs underwent HIPEC treatment with bromelain, cisplatin, and mitomycin C (MMC) at 37 °C and 42 °C with and without bromelain pretreatment.

**Results:**

From October 2020 to May 2023, 16 specimens were collected from 13 patients with low-grade (12/16, 75%) and high-grade AC (4/16, 25%). The mucin-depleting effects of bromelain were most significant in combination with N-acetylcysteine (NAC) compared with bromelain (47% versus 10%, *p* = 0.0009) or NAC alone (47% versus 12.8%, *p* = 0.0027). Bromelain demonstrated > 31% organoid viability reduction at 60 min (*p* < 0.001) and > 66% in 48 h (*p* < 0.0001). Pretreatment with bromelain increased cytotoxicity of both cisplatin and MMC HIPEC conditions by 31.6% (*p* = 0.0001) and 35.5% (*p* = 0.0001), respectively. Ki67, CK20, and MUC2 expression decreased after bromelain treatment; while increased caspase 3/7 activity and decreased Bcl-2 (*p* = 0.009) and Bcl-xL (*p* = 0.01) suggest induction of apoptosis pathways. Furthermore, autophagy proteins LC3A/B I (*p* < 0.03) and II (*p* < 0.031) were increased; while ATG7 (*p* < 0.01), ATG 12 (*p* < 0.04), and Becline 1(*p* < 0.03), expression decreased in bromelain-treated PTOs.

**Conclusions:**

Bromelain demonstrates cytotoxicity and mucolytic activity against appendiceal cancer organoids. As a pretreatment agent, it potentiates the cytotoxicity of multiple HIPEC regimens, potentially mediated through programmed cell death and autophagy.

**Supplementary Information:**

The online version contains supplementary material available at 10.1245/s10434-024-15355-0.

Cytoreductive surgery with heated intraperitoneal chemotherapy (CRS/HIPEC) has become the standard of care for patients with peritoneal dissemination of appendiceal primaries.^1–3^ While patients with mucinous appendiceal tumors experience improved survival compared with those with non-mucinous tumors, mucin has been hypothesized to serve as a physical barrier to drug delivery for HIPEC therapy.^4–7^ Currently, there are no Food and Drug Administration (FDA)-approved mucolytics in the treatment of appendiceal cancer (AC) in the USA. Physically removing mucin during CRS/HIPEC has served as the primary modality for mucin debulking with minimal effect on limiting further mucin production.

Bromelain is a pineapple stem-derived extract, and its mucolytic activity has generated interest in treatment of AC through hydrolysis of the glycosidic bonds found in mucin.^8,9^ When combined with N-acetylcysteine (NAC), it results in near complete dissolution of mucinous ascites.^9,10^ Further, it has been shown to downregulate markers associated with proliferation and chemoresistance as well as display innate cytotoxic activity in gastrointestinal (GI) tumors, including those with peritoneal dissemination.^11–13^

We have previously shown the feasibility of patient-derived tumor organoid (PTO) research applications in rare and common cancers, including appendiceal cancer.^14–18^ In this study, we analyzed the activity of bromelain against appendiceal cancer organoids. We hypothesized that bromelain could serve as a pretreatment agent to traditional intraperitoneal perfusates utilized in HIPEC therapy, thereby reducing the mucinous burden, and improving tumor contact with drug. We also utilized PTOs to study the possible mechanisms responsible for the activity of bromelain.

## Patients and Methods

### Tumor Procurement and Processing

Tumor specimens were obtained from patients with AC undergoing CRS/HIPEC at Wake Forest Baptist Medical Center between October 2020 and May 2023. Specimens were obtained in accordance with institutional guidelines and under an approved institutional review board (IRB) protocol. Owing to the small study population, race/ethnicity data are not presented. Specimens were placed in Roswell Park Memorial Institute (RPMI) medium and transferred to the Wake Forest Organoid Research Center (WFORCE) for tissue processing within 2 h from surgical resection.

Upon arrival, specimens were washed in two 5 min cycles with a Dulbecco’s phosphate-buffered saline (DPBS) solution containing 100 U/mL penicillin/streptomycin, 5 μg/mL gentamicin (G1272, Sigma Aldrich, St. Louis, MS), and 5 μg/mL amphotericin B (A2942, Sigma). The tumor specimen was minced finely, removing fat and necrotic tissue. Each gram of minced tissue was placed into a 15 ml conical tube containing Dulbecco’s modified Eagle’s medium (DMEM)/F12 advanced medium containing 100 U/mL penicillin/streptomycin, 5 μg/mL gentamicin, 5 μg/mL amphotericin B, 100,000 cytidine deaminase (CDA) units/mL collagenase HA (001-1050; VitaCyte, Indianapolis, IN), 22,000 narcissus pseudonarcissus agglutinin (NPA) units/mL BP Protease (003–1000; VitaCyte), 50 U/ml DNase I (07900; StemCell Technologies, Cambridge, MA), and 20 mM N-acetyl L-cysteine (A9165; Sigma), with the total volume of 3 mL solution per gram of tissue. Tubes were placed on a mixing rack for 90 min at 37 °C. After digestion, 5 mL of cold RPMI with 10% fetal bovine serum (FBS) was added to neutralize enzymatic activity. The digested tissue mixture was filtered using a 100 uM sterile filter and centrifuged. BD Pharm LyseTM (555899; BD Biosciences) was used to lyse red blood cells according to the manufacturer’s protocol. After red blood cell lysis, cells were resuspended in DMEM/F12 with 10% FBS and counted using a NucleoCounter® NC-200™ (Chemometec, Denmark).

### Organoid Fabrication and Organoid Culture

Organoids were fabricated similarly to previously published methods.^14,19^ Briefly, 10 million cells/ml were resuspended in hydrogel comprising of 3 mg/ml methacrylated collagen (PhotoCol; Advanced Biomatrix) and thiol-modified hyaluronan/heparin (Heprasil; Advanced Biomatrix) in a 3:1 volume ratio. Then, 5 μL of the hydrogel-cell suspension was deposited into each well of 48 well plates coated with cured polydimethylsiloxane (PDMS) to create dome cultures. After seeding, hydrogel was photo-crosslinked by exposure to ultraviolet light and PTOs were cultured in 500 μL media containing advanced DMEM-F12 with 5% FBS, 1% penicillin-streptomycin, 1% L-glutamine, 50 ng/mL EGF (PHG 0313; Thermo Fisher Scientific), and 10 uM Y-27632 (S1049, Selleckchem, USA), with media changes twice per week. PTOs were maintained in culture for 7 days prior to the treatment.

### Naxos 5 Immortalized Appendiceal Cancer Cell Line Organoids

Naxos 5 appendiceal cancer is a signet ring AC cell line, generated by the Wake Forest Organoid Research Center (WFORCE) and the Wake Forest Cellular Engineering Shared Resource. Naxos 5 organoids were fabricated and cultured as described above with little modification. Naxos 5 cell line and organoids were cultured in medium containing DMEM advanced plus basal medium (Sigma, cat # SCM16) with 1X antibiotic-antimycotic (Fisher, cat # 15240062), 10 mM HEPES (Invitrogen, cat # 15630-056), 1X L-Glutamine (Gibco, cat# 25030081), 1X supplement N2 (Gibco, cat # A1370701), 1X supplement B27 (Gibco, cat # 17504-44), 5 mM nicotinamide (Sigma, cat # N0636), 1.25 mM N-acetylcysteine (Sigma, cat # A9165), 500 nM SB202190 (Selleckchem, cat # S1077) , 500 nM A83-01 (Selleckchem, cat # S7692), 100 ng/ml Noggin (Peprotech, cat # 120-10C), 5 ng/ml EGF (Peprotech, cat # AF-100-15), 20 ng/ml FGF 10 (Peprotech, cat # 100-26), 5 ng/ml FGF 7 (Peprotech, cat # 100-03), 250 ng/ml R-spondin 1 (Peprotech, cat # 120-38), and 10 uM rock 1 inhibitor Y-27632 2HCL (Selleckchem, cat # S1049).

### Drug Preparation

Pharmaceutical grade bromelain and N-acetylcysteine were provided by Mucpharma Ltd (Australia). Mitomycin C (S8146, Selleckchem), cisplatin (S1166, Selleckchem), and doxorubicin (E2516, Selleckchem) were used in HIPEC regimens. MMC, Cisplatin, and doxorubicin stock solutions were prepared at concentrations of 10 mM according to manufacturer’s instructions and diluted fresh in culture media for final doses of 10 μg/mL, 10 uM, and 0.5 uM, respectively, before use. For each experiment, bromelain stock solution (5 mg/mL in culture media) was freshly prepared and diluted to achieve the final treatment concentration of 600 μg/ml.

### Ex Vivo Mucin Dissolution Study

In total, 20 g of freshly procured tumor produced mucin from patients was equally distributed in four groups. Mucin was treated with either 20 mL of bromelain (600 μg/ml in DPBS), 3% *N*-acetylcysteine in DPBS, or a combination of bromelain and N-acetylcysteine, with a DPBS control group. Bromelain and *N*-acetylcysteine doses were derived from previous phase I bromelain studies and the current phase II study.^20,21^ Mucin-containing dishes were incubated for 3 h at 37 °C on an orbital shaker. Residual mucin was then collected and weighed. To calculate the percentage of mucin lost after treatment, we used the following formula: % mucin disintegration = (pre digestion mucin weight − post digestion mucin weight)/pre digestion mucin weight.

### Bromelain Treatment at 37 °C

After 7 days in culture, media was removed from the PTOs and replaced with 500 μL of fresh medium containing 600 μg/ml bromelain in addition to nontreated comparison PTOs. PTOs were cultured at 37 °C for 60 min, 120 min, 48 h, and 72 h. After each time point, PTOs were harvested to assess bromelain treatment effect.

### HIPEC Treatments

On day 7, culture medium was aspirated and treatment medium containing either bromelain, cisplatin, MMC, or doxorubicin was added. PTOs were incubated for 60 min at 42 °C. After treatment, media was removed from the PTOs, washed in culture media once, and fresh culture media was added to each well.

To determine whether pretreatment with bromelain sensitizes PTOs to HIPEC, sequential treatment was performed. More specifically, PTOs were first treated with bromelain for 60 min, washed, and treated again with either MMC, cisplatin, and doxorubicin for an additional 60 min at 37 °C and 42 °C in parallel treatment groups. Between treatments, PTOs were washed to remove treatment medium and further cultured for 72 h post-treatment at 37 °C. PTOs were then assessed to determine the antitumor activity of single and sequential treatments. The control PTOs were cultured in the same drug-free medium containing the same concentration of the drug solvent as the treatment PTOs.

### Tumor Organoid Viability Assessment LIVE/DEAD Imaging

Following 72 h of treatment, PTO viability was assessed using the Invitrogen Live/Dead viability/cytotoxicity kit (L3224; Invitrogen, Carlsbad, CA). Fluorescent images were acquired using a Leica TCS LSI macro confocal microscope (Leica, Wetzlar, Germany); cells expressing green fluorescence were considered viable while red indicated cell death.

### 3D CellTiter Glo Luminescent Cell Viability Assay

After 72 h of drug treatment, ATP quantification for viability was performed. Briefly, PTOs were transferred to 96-well Costar White polystyrene assay plates (3912; Corning, NY) and the three-dimensional (3D) CellTiterGlo luminescent cell viability assay (G968B; Promega, Madison, WI) was performed according to manufacturer’s instructions. Luminescence was read on a VariskanTM LUX multimode microplate reader (ThermoFisher Scientific).

### Apoptosis Assay

To determine Apoptosis and Caspase 3/7 activity, the NucView® 488 and CF®594 Annexin V Dual Apoptosis Assay Kit for Live Cells (30067, Biotium, Freemont, CA) and NucView® 488 & MitoView 633 Apoptosis assay kit (30062, Biotium, Freemont, CA) were used according to the manufacturer’s recommendation. Fluorescent images of whole-mount PTOs were acquired using a Leica TCS LSI macro confocal microscope.

For real-time detection of changes in mitochondrial membrane potential and Caspase 3/7 activity in Naxos 5 organoids, NucView® 488 and Mitoview® 633 reagents were used according to the manufacturer’s recommendation. Organoid images were acquired using Leica TCS LSI macro confocal microscope.

### Histology

Organoids were fixed for histology at the end of the drug study in 4% buffered paraformaldehyde (PFA) for 1 h to prepare for paraffin embedding. Hematoxylin and eosin (H&E) staining was performed on sectioned slides prior to the immunofluorescence staining. Before immunofluorescence staining, slides underwent antigen retrieval in a pH 6.0 citrate buffer solution. Slides underwent protein blocking using Dako Protein Blocker solution for 60 min. After blocking, sections were incubated with primary antibodies overnight at 4 °C in a humidified chamber using cytokeratin 20 (ab76126, Abcam), Ki-67 (ab16667, Abcam), and mucin 2 (ab11197, Abcam). All primary antibodies were diluted in Dako antibody diluent and used in a 1:50 dilution. After primary antibody incubation, washes were performed followed by application of appropriate species reactive secondary antibodies labeled with Alexa Fluor 488 or Alexa Fluor 594 antibodies (Biotium) for 1 h at 1:1000 dilution. After washing, sections were mounted with Prolong Diamond Antifade Mountant with 4′,6-diamidino-2-phenylindole (DAPI; P36971, Invitrogen). Sections were imaged using an Olympus BX-63 upright fluorescent microscope (Olympus, Tokyo, Japan).

### Autophagy and Apoptosis Analysis

Additional autophagy and apoptosis analysis was conducted to include organoid lysates and western blotting, included in the Supplementary Data.

### Statistical Analysis

**Fig. 1:**
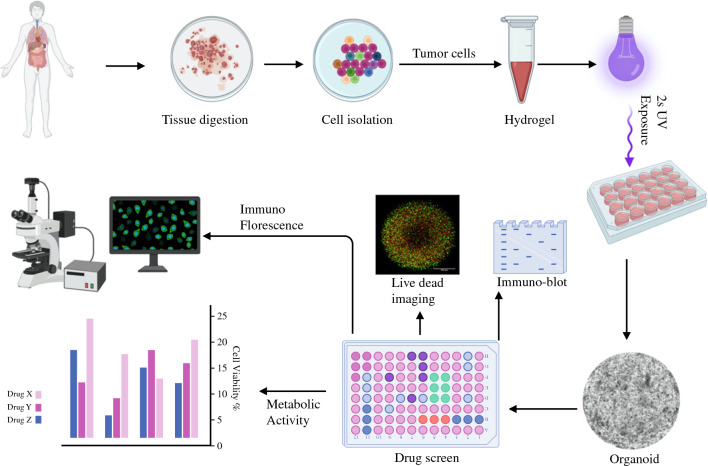
Schematic workflow of organoids fabrication from appendiceal tumor specimens and downstream analysis. Day 1 concludes with tumor cell isolation and encapsulation into the ECM-based hydrogel for organoid fabrication. After one week of culture, organoids are treated with HIPEC regimens for 72 hours, followed by analysis

Viability data are expressed as mean ± standard deviation. Each treatment group consisted of five organoids for viability analysis and three organoids for immunoblotting. Adenosine triphosphate (ATP) values were standardized to a control value of 100 for ease of interpretation. We performed unpaired Student’s *t*-tests between two groups of interest. Statistical analysis was performed with GraphPad Prism (GraphPad Software Inc., USA). The ROUT method in GraphPad was used with a Q = 1% to remove outlier values for analysis of pooled data. A *p*-value of < 0.05 was used for statistical significance (Fig. 1).

## Results

### Clinical Information and Biofabrication of Appendiceal Cancer Organoids

A total of 16 tumors from 13 patients were enrolled in this study; 10 patients (77%) had low-grade appendiceal primaries (LGA) while 3 patients (23%) had high-grade appendiceal primaries (HGA). Each of the patients with HGA received neoadjuvant FOLFOX-based regimen including one patient with LGA. Two patients had undergone prior curative intent surgery (Table 1). Testing was performed successfully on 16/16 (100%) PTO sets for comparison of HIPEC efficacy.

**Table 1:** Descriptive information: histology, anatomic site and prior treatment

Tumor	Grade	Site	Neoadjuvant chemotherapy	Prior surgery
1	HGA	Pelvic stripping	FOLFOX	CRS/HIPEC
2	LGA	Omentum	None	None
3	HGA	Appendix	FOLFIRINOX	None
4	LGA	Omentum	None	None
5	LGA	Omentum	None	None
6	LGA	Peritoneal Tumor Deposit	None	None
7	LGA	Omentum	None	None
8	LGA	Omentum	none	None
9A	HGA	Appendix	mFOLFOX/Avastin	Diagnostic Laparoscopy
9B	HGA	R ovary		
10	LGA	Omentum	None	None
11	LGA	Ovary	None	None
12	LGA	Small Bowel	None	CRS/HIPEC
13A	LGA	Pelvic Stripping 1	FOLFOX	CRS/HIPEC
13B	LGA	Pelvic Stripping 2		
13C	LGA	Pelvic Stripping 3		

Thirteen patients contributed 16 specimens. Three patients had high grade appendiceal primaries (HGA) while ten were low grade (LGA). Due to the small study population, race/ethnicity data is not presented

### Mucin Degradation

**Fig. 2:**
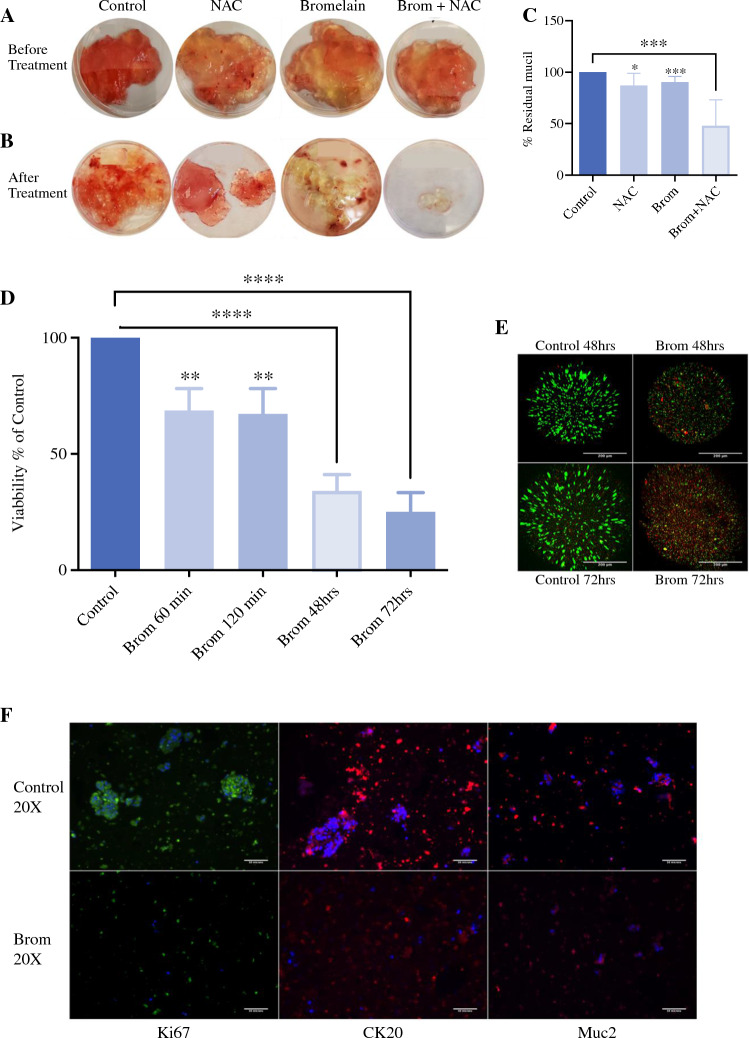
Mucolytic and cytotoxic effects of bromelain **A** Untreated mucin from ovarian metastasis from a low grade appendiceal cancer **B** Same mucin, 3 hours after treatment with 3% NAC, 600 mg/ml bromelain, bromelain and NAC (Brom + NAC), or PBS/untreated (control). **C** Pooled analysis of mucin degradation Plot (N=7) shows the percentage of residual mucin post-treatment under different conditions. P values of bromelain *p* < 0.0006, NAC *p* < 0.012, Brom+NAC *p* < 0.0001 compared to the control. **D** Pooled analysis (n=11) of ATP viability assay of organoids after treatment with 600 ug/ml bromelain for 60 min, 120 min, 48 h, and 72 h at 37 °C. (Mean ± SD, n = 11; **p < 0.01, ****p < 0.0001). AC PTO viability after 60 and 120 min of bromelain treatment was decreased by 31.2% and 32.2%, respectively. AC PTO Viability was further decreased by 65.9% after 48 hours and 74.8% after 72 h. **E** Corresponding Live/Dead stain at 48 and 72 hours showing > 43% and 80.2% decrease in viability, respectively. **F** show the AC PTO Immunohistochemical images of Ki67 (Alexa 488), ii. CK20 (Alexa 594), and iii. MUC2 (Alexa 594) following bromelain treatment. Nuclei were stained with DAPI

To analyze the degradation of gross mucin in appendiceal tumors in normothermic conditions, 20 g of mucinous tissue, removed from a low-grade ovarian metastatic lesion (patient 11), were treated with three different mucolytics: (1) bromelain 600 μg/ml, (2) N-Acetyl-Cysteine (NAC) 3% (reduces the heavily cross-linked mucus glycoproteins, and (3) bromelain combined with NAC (Fig. 2A). After 3 h of treatment at 37 °C (Fig. 2B), the mucinous tissue was weighed, and the percentage of remaining mass was calculated. In pooled analysis (*N* = 7), bromelain-NAC resulted in 52% (*p* < 0.0001) mucin degradation compared with 10% (*p* = 0.0006) mucin degradation achieved by bromelain or 12.8% (*p* = 0.012) achieved by NAC alone (Fig. 2C). Testing of organoids with combined bromelain NAC was not pursued owing to NAC-induced disintegration of the collagen-Heprasil hydrogel, resulting in disruption of gel structural integrity.

### Bromelain Cytotoxicity Dependency on Duration of Exposure

The cytotoxic effects of bromelain at 600 μg/mL were examined in AC PTOs. Pooled analysis of 11 samples demonstrated a statistically significant (31.16%, *p* < 0.001) reduction in cellular viability after 60 min of treatment with minimal additional cytotoxicity at 120 min. However, cytotoxicity progressively increased to a 65.91% (*p* < 0.0001) reduction in cellular viability after 48 h of treatment (Fig. 2D). Qualitative live dead images of bromelain treatment after 48 h and 72 h also reflect the significant cell death in bromelain-treated PTO compared with control (Fig. 2E).

Next, we analyzed the expression of proliferation and tumor identification markers in AC tumor organoids. Bromelain treatment reduced Ki67 expression in AC PTOs, suggesting an antiproliferative activity (Fig. 2F). Furthermore, the total expression of cytokeratin 20 (CK20) in the appendiceal cancer organoids decreased upon bromelain treatment, indicating a reduction in the tumor cell population (Fig. 2F).

To validate the results of bromelain on mucin degradation, we analyzed the expression of Mucin 2 (MUC2) in appendiceal cancer organoids. After 48 h of bromelain treatment, cellular MUC2 expression was greatly diminished, suggesting both a reduction in tumor cells and a loss in mucin production (Fig. 2F).

### Bromelain Pretreatment Effect in Appendiceal Cancer PTOs

**Fig. 3:**
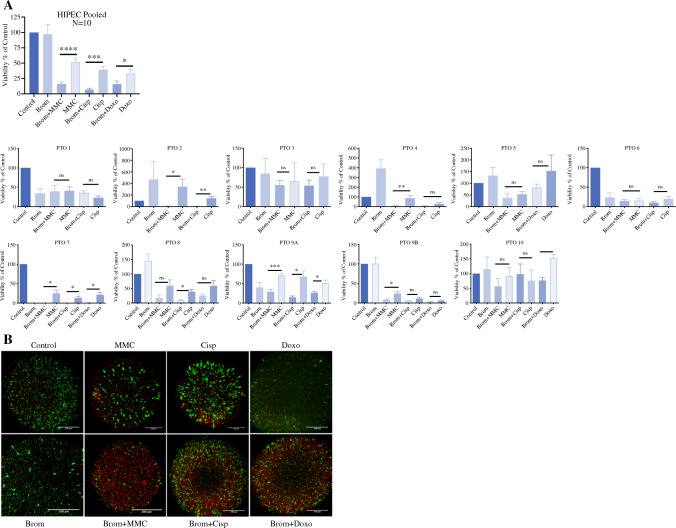
Bromelain potentiates HIPEC effect when used as a pretreatment agent at 42 °C. **A** Pooled analysis (N=10) of ATP viability of AC PTOs after 1 hour of treatment with bromelain ± 1 additional hour of HIPEC with MMC, Cisplatin, and doxorubicin (42 °C). ATP viability data demonstrates synergistic action when PTOs are pre-treated with bromelain before HIPEC perfusates (Mean ± SD, N = 10; MMC vs Brom+MMC *p* < 0.0001, Cisplatin vs Brom+Cisplatin *p* < 0.0001, doxo vs Brom + doxo *p* < 0.02). PTOs 1-10 represent individual AC patients. **B** Representative Live/Dead stain imaged with confocal microscopy. Green color live cells. Red color dead cells, respectively. Scale bar 200 um

To test the effect of pretreatment with bromelain on HIPEC regimens, AC PTOs were treated for 1 h with bromelain followed by an h of mitomycin-C (MMC), cisplatin (cisp), or doxorubicin (doxo) at 42 °C. Significant heterogeneity in post-treatment cytotoxicity was observed among patients, as depicted in Fig. 3A (PTOs 1–10).

Pooled analysis of all patients (*N* = 10), organoids pretreated with bromelain followed by MMC, cisplatin, and doxorubicin show a significant reduction in tumor cell viability compared with control, untreated organoids (Fig. 3A). The combination of bromelain and MMC resulted in 83.72% viability reduction compared with 48.26% viability reduction with MMC alone (*p* < 0.0001 versus untreated controls). Similarly, cisplatin pretreated with bromelain resulted in 92.6% versus 60.6% viability reduction when compared with cisplatin as a single agent (*p* < 0.0001). Doxorubicin combined with bromelain reduced cell viability by 83.8% versus 66.5% reduction observed with doxorubicin treatment alone compared with untreated controls (*p* < 0.02). Live dead staining qualitatively demonstrated reduced cell viability in AC PTOs treated with bromelain and HIPEC perfusates compared with bromelain or perfusates alone (Fig. 3B).

**Fig. 4:**
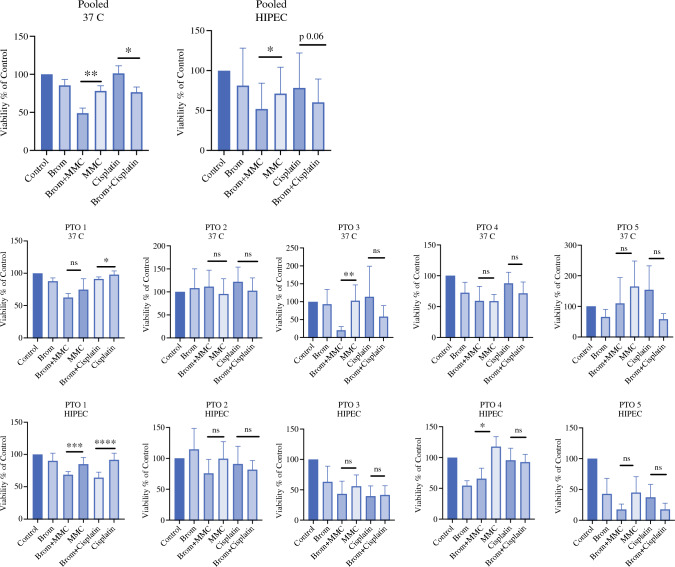
The effect of bromelain on ATP viability of AC PTOs after 1 hour of treatment with bromelain followed by 1 hour of MMC or Cisplatin at 37 °C and 42 °C. PTOs are from 5 different specimens with PTOs 3 to 5 originating from spatially distinct sites of the same patient. Upper Row: Pooled (N = 5) ATP viability at 37 °C demonstrates synergistic action when PTOs are pre-treated with bromelain (Mean ± SD, MMC vs Brom+MMC *p* < 0.002, Cisplatin vs Brom+Cisplatin *p* < 0.03), that is less prominent when bromelain is heated at 42°C (MMC vs Brom+MMC *p* < 0.02, Cisplatin vs Brom+Cisplatin *p* < 0.06). Middle and lower row: ATP viability at 37 and 42 °C based on specimen

We also compared differential treatment responses at 37 °C and 42 °C from three individual patients (Fig. 4). Patient 13 from this group had three spatially distinct tumors (PTO 3–5, Fig. 4). We tested PTOs generated from each of the three sites with 60 min. of bromelain followed by 60 min. of MMC or cisplatin at 37 °C and 42°C. Hyperthermia did not individually improve the effects of bromelain pre-treatment at any of the three sites. However, pooled analysis revealed improved cytotoxicity of MMC and cisplatin when pre-treated with bromelain at both 37 °C and 42 °C.

At 37 °C, the combination of bromelain and MMC reduced AC PTO cell viability by 50.8% versus 21.7% reduction obtained by MMC alone compared with the control (*p* < 0.002, *n* = 3). Cisplatin alone at 37 °C did not affect viability while cisplatin combined with bromelain reduced viability by 23.4% (*p* < 0.03, *n* = 3).

**Fig. 5:**
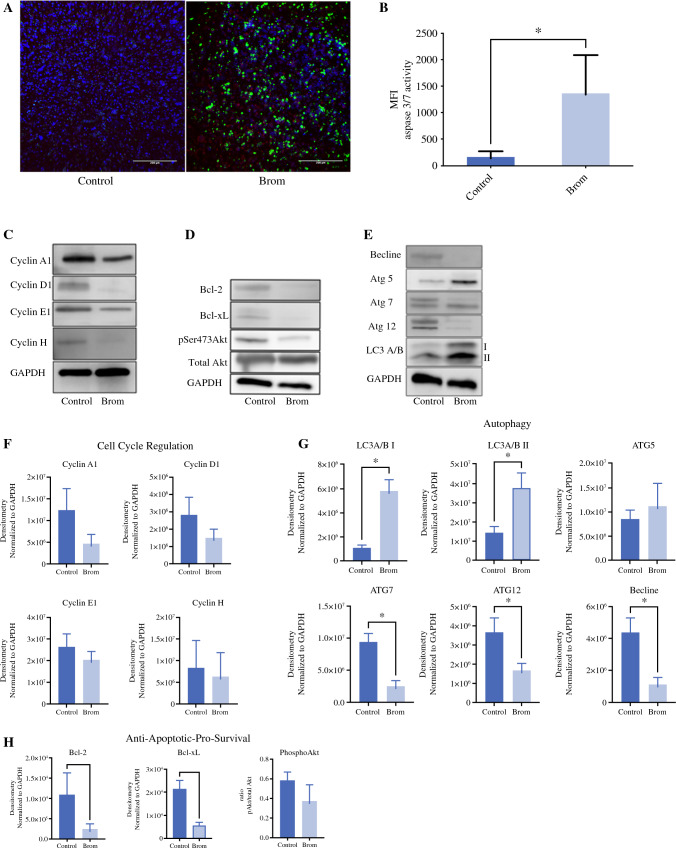
Bromelain induces apoptosis in appendiceal PTOs observed through caspase activity and autophagy/apoptosis protein expression analysis. **A** Annexin V expression is in red, and caspase 3/7 activity expression is in green. Nuclei were stained with DAPI. **B** Pooled Caspase 3/7 activity from three individual patients. Bromelain-treated AC organoids show increased 3/7 activity compared to the control (*p* < 0.04, n = 3). Confocal images from A were quantitated in ImageJ. Bars represent the Mean fluorescence intensities (MFI) of Caspase 3/7 activity. **C/F:** Protein expression and densitometry shows trending inhibition of cyclin A2, Cyclin D1, Cyclin E1, and Cyclin H in bromelain-treated organoids (n=3). Densitometry of cell cycle protein expression normalized to GAPDH. **D/H** Densitometry of anti-apoptotic and pro-survival protein expression in Figure 5D. Figure 5H densitometry analysis shows significant inhibition of Bcl-2 *p* < 0.009 and Bcl-xL *p* < 0.01, n=3. **E/G:** represent the densitometry analysis of autophagy protein expression in Figure 5E. Densitometry analysis of autophagy protein expression shows that ATG7 *p* < 0.01, ATG 12 *p* < 0.04, and Beclin-1 expression *p* < 0.03 inhibit in bromelain-treated organoids. Expression of LC3 A/B I *p* <  0.01 and LC3 A/B II *p* < 0.03 both are increasing in bromelain-treated organoids compared to the control (n = 3)

At 42 °C, pretreatment with bromelain followed by MMC resulted in 48.1% viability reduction versus 28.7% (*p* < 0.007, *n* = 3) obtained by MMC alone. Cisplatin treatment alone at 42 °C resulted in 21.54% viability reduction compared with 39.6% (*p* < 0.06 trending to significance, *n* = 3) reduction observed with bromelain and cisplatin. These results suggest that sequential treatment with bromelain and HIPEC perfusates may enhance overall antitumor cell activity but may be limited by site-specific tumor heterogeneity, irrespective of perfusate temperature (Fig. 5).

### The Antitumor Activity of Bromelain is Mediated via Apoptosis and Autophagy

To explore potential mechanisms underlining the antitumor activities of bromelain, we analyzed its effect on tumor cell apoptosis, cell cycle regulation, and autophagy pathways (Supplementary Data).

### Effect of Bromelain Pretreatment on Naxos 5 Appendiceal Cancer Cell Line Organoids

**Fig. 6:**
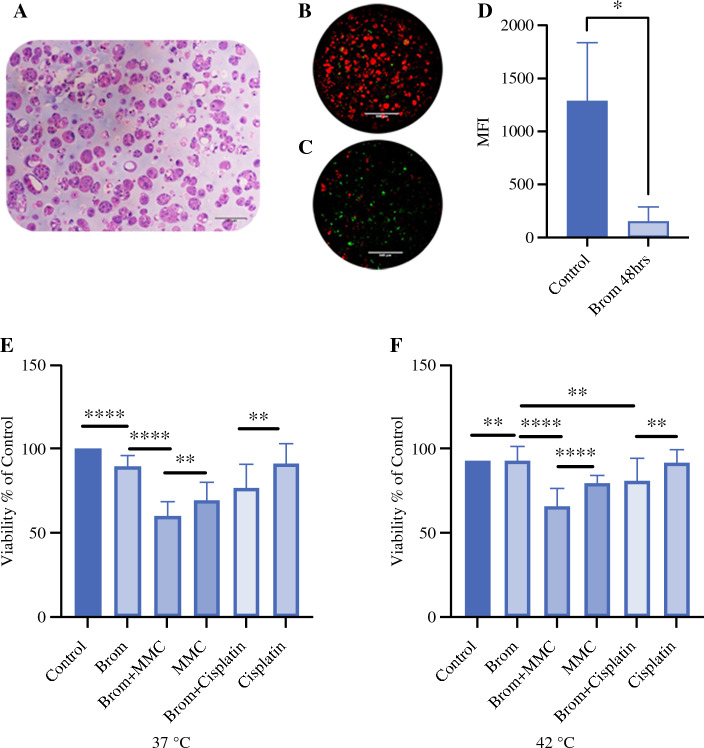
Effect of bromelain on Naxos 5 appendiceal tumor cell line organoids. **A** signet ring morphology on 10X H&E of Naxos 5 appendiceal cancer cell line organoids. **B**, **C** Confocal images of Naxos 5 organoids stained with live Mitoview and Caspase 3/7 substrate. Figure 6B represents organoids treated with bromelain for 48 hrs. Figure 6C control demonstrates untreated organoids. Red fluorescence in Figure 6B-C indicates intact mitochondrial membrane potential in organoids, and green fluorescence represents the caspase 3/7 activity. **D** Mean fluorescence intensity (MFI) of red fluorescence representing intact mitochondrial membrane potential (**B**, **C**) in untreated vs bromelain-treated organoids. Bromelain treatment significantly inhibits mitochondrial membrane potential (87.87% compared to the control) with *p* < 0.02 control vs bromelain (n = 3). **E**: Pooled ATP viability of Naxos 5 organoids after 1 hour of treatment with bromelain ± 1 additional hour at 37 °C with MMC and Cisplatin. ATP viability data reveals synergistic action when organoids are pre-treated with bromelain at 37 °C (Mean ± SD, n = 3; MMC vs Brom + MMC *p* < 0.007, Cisplatin vs Brom + Cisplatin *p* < 0.003. **F** HIPEC 42 °C Pooled (N = 3) ATP viability of Naxos 5 organoids after 1 hour of treatment with bromelain ± 1 additional hour of HIPEC with heated MMC, and Cisplatin (42 °C). MMC vs Brom + MMC *p* < 0.0001, Cisplatin vs Brom+Cisplatin *p* < 0.006

Naxos 5 is a signet ring cell appendiceal adenocarcinoma cancer cell line created by the Wake Forest Organoid Research Center (WFORCE) and the Wake Forest Cellular Engineering Shared Resource using hTERT methodology. Organoids fabricated from this cell line maintain a signet ring cell arrangement in H&E staining, as shown in Fig. 6A (10× magnification).

Bromelain treatment in these organoids disrupts the mitochondrial membrane potential, a hallmark for apoptosis, as evident from low mitochondrial membrane staining in organoids treated with bromelain compared with the untreated control organoids (Fig. 6B and C).

Figure 6D represents the mean fluorescence intensity (MFI) of red fluorescence in images 6B–C. Bromelain treatment significantly inhibits mitochondrial membrane potential (87.87%, *p* < 0.02, *n* = 3) compared with the control, suggesting bromelain-induced cell death in these organoids.

We also used these organoids to study the effect of bromelain on organoid viability in combination with MMC and cisplatin under normothermic (37 °C) and HIPEC (42 °C) conditions (Fig. 6E and F). On a pooled basis (*N* = 3) under normothermic conditions (Fig. 6E), bromelain treatment resulted in 10.6% viability reduction compared with untreated controls (*p* < 0.0001). Organoids treated with MMC and bromelain resulted in 40% viability reduction compared with 30.7% (*p* < 0.007) in MMC alone. Similarly, cisplatin resulted in 8.7% viability reduction compared with cotreatment with cisplatin and bromelain (23.2% viability reduction, *p* < 0.003).

On a pooled basis (*N* = 3) under HIPEC conditions (Fig. 6F) bromelain showed a similar viability inhibition pattern compared with normothermia, reducing post-treatment viability by 7% compared with controls (*p* < 0.011). MMC reduced viability by 20.4% when treated independently, while bromelain combined with MMC resulted in a 34% viability reduction compared with control (*p* < 0.0001). Finally, bromelain combined with cisplatin resulted in 19% viability reduction in Naxos 5 organoids compared with 8.2% viability reduction in cisplatin-treated PTOs (*p* < 0.006).

## Discussion

Surgical debulking of mucin prior to HIPEC perfusion is limited by residual mucin deposits. This theoretically impedes tumor–drug contact during HIPEC and thereby limits treatment efficacy and ultimately, survival. Furthermore, extensive mucin involvement may erroneously guide intraoperative decisions toward unnecessary organ resections. Currently, there are no mucin-specific treatment options for patients with AC. Research on AC mucin has largely focused on marker expression^22–24^ and characterization.^25^ This current study is the first to examine perfusion with bromelain in a preclinical organoid model as a potential component of CRS/HIPEC procedures performed for mucinous appendiceal neoplasms with peritoneal dissemination.

The mucin-specific experiments in this study demonstrated significant mucolytic activity with about 50% reduction in fresh mucin weight after exposure to both bromelain and NAC in just 60 min, which is in line with previous experiments at similar concentrations.^9^ Currently, one phase I clinical study by Valle et al.^20^ examined BromAc® activity in patients with unresectable AC. An objective response was seen in approximately 73% of sites treated (30/41) and 85% of patients (17/20) after intraperitoneal or intratumoral injection. The results have led to the development of an upcoming phase II clinical trial later this year.^21^ This may have great utility especially in patients undergoing repeat CRS/HIPEC where mucin appears to have undergone varying degrees of fibrosis, and thus, is more difficult to be addressed.

In addition to the mucolytic properties of bromelain, we were interested to see if bromelain could serve as a pretreatment agent to HIPEC therapy. Our experimental treatment outline involved pre-treatment of PTOs with bromelain for 60 min prior to an additional 60 min of treatment with traditional HIPEC drugs, thereby maintaining the total 2 h perfusion time we utilize clinically. We rationalized that bromelain pretreatment would improve tumor-drug contact through its ability to degrade mucin leading to improved chemotherapy efficacy through its effect on arresting cell cycle, decreasing expression of Ki-67, and activating apoptotic and autophagy pathways (Supplementary Data). Overall, this treatment model demonstrated improved cytotoxicity compared with 120 min of HIPEC alone. As expected, we observed heterogeneity not only among patients but also among treatment sites from the same patient. Heterogeneity and bromelain sensitivity are part of an ongoing study to further characterize these initial results. Further, examining these effects under pooled analysis may generate improved prediction of overall response clinically.

While we believe this study provides further evidence that bromelain may play a role in AC treatment, there are a few limitations. The study cohort was limited to 13 patients and would benefit from continued accrual to determine clinically relevant statistical differences between treatment groups. Most patients contributed a single tumor site to the study which limits the ability to account for possible treatment heterogeneity between multiple tumor sites in the same patient. We focused this study on appendiceal cancer from patients who were undergoing their first CRS/HIPEC and thus we did not examine the effects of bromelain’s ability to degrade fibrotic mucinous samples in patients with long standing or recurrent disease. The safety of bromelain on bowel anastomosis has been examined separately but was not part of this study.

## Conclusions

Bromelain displays mucolytic activity against fresh AC-derived mucin that is enhanced by NAC as well as cytotoxic activity against both patient and cell line derived AC organoids. These characteristics make bromelain an intriguing pre-HIPEC treatment option for patients with peritoneal carcinomatosis from AC, as it enhances tumor–drug contact by inducing mucolysis and augments cytotoxicity of current HIPEC agents.

### Supplementary Information

Below is the link to the electronic supplementary material.

Supplementary file1 (DOCX 28 KB)
