# Supplementary material for: Enhancing the Efficacy of HIPEC Through Bromelain: A Preclinical Investigation in Appendiceal Cancer
Source: Ann Surg Oncol. 2024 May 4;31(8):5377–89. doi: 10.1245/s10434-024-15355-0 (PMC11236891; doi:10.1245/s10434-024-15355-0)
Supplement: Supplementary file 1 — Supplementary file1 (DOCX 28 KB) [file 10434_2024_15355_MOESM1_ESM.docx]

**Enhancing the Efficacy of HIPEC through Bromelain:**

**A Preclinical Investigation in Appendiceal Cancer**

Supplementary Data on Apoptosis and Autophagy

**Methods**

***PTO lysates***

To analyze protein lysate, PTOs were collected after drug treatment and washed in cold DPBS twice to remove any residual media before protein extraction. Control and bromelain treated PTOs (n=4) were lysed using QIGEN Tissue Lyser LT. Briefly, the PTOs were transferred to propylene tubes; in each tube, a 5mm prechilled QIAGEN stainless steel bead was added (6989, QIAGEN) along with cold 400 ul RIPA buffer (89900, ThermoFisher) and a protease and phosphatase inhibitor cocktail (PPC1010, Sigma). Tubes were secured in a Tissue lyser and lysed for one minute. After lysis, tubes were immediately transferred to ice and incubated for fifteen minutes. PTOs crude extract was centrifuged in the cold for thirty minutes at 12,000g. The supernatant containing protein lysate was collected and transferred to clean tubes. Protein lysate concentration was determined using the Qubit Protein Assay kit (Q33211, Q33212 Invitrogen) and read on Qubit 4-Fluorometer (Q33226, Invitrogen).

***Western Blotting***

To assess PTO western blot expression, 45 ug of total protein from control and bromelain treated was used. Samples were mixed with 2× Laemmli sample buffer (161-0737-BioRad) and incubated for 5 minutes at 95°C. Proteins were separated on 4-20% linear gradient SDS gels (BioRad) under constant voltage (100V) with 1X Tris-Glycine SDS running buffer (1610732, BioRad). Separated proteins were transferred to a polyvinylidene fluoride membrane (PVDF) (162-0175-BioRad) using a cold transfer buffer (1610734, BioRad) with 20% methanol at 100V for 60 minutes. Membranes were blocked in Superblock T20 buffer (37536, ThermoFisher) at room temperature for sixty minutes and then incubated with primary antibodies at 4°C overnight in block buffer. The membranes were washed three times, 10 minutes each, in 1X washing buffer (1706435, BioRad) with 0.05% tween 20. After washing, a horseradish peroxidase-conjugated secondary antibody (1: 1000) was added to the block buffer at room temperature for 60 minutes. Following three 10-minute washes, membranes were visualized by SuperSignal™ West Atto Ultimate Sensitivity HRP Substrate kit (A38554, ThermoFisher) and detected by iBright system (ThermoFisher). The images were digitized with the iBright system and quantitated with Fiji software. For total protein loading, membranes were stripped using stripping buffer (46430, ThermoFisher) for 15 minutes at room temperature with rocking. After stripping, the membranes were washed three times in washing buffer, as described above. GAPDH antibody was added to the washed membranes for loading control, and images were acquired as described above.

Primary antibodies and their dilutions are as follows:

Beclin 1 (D40C5-CellSignaling) 1:1000, BcL-XL (54H6-Cell Signaling) 1:1000, Bcl-2 (D55G8-CellSignaling) 1:1000, Cyclin H (2927T-CellSignaling) 1:500, Cyclin E1 (HE12-CellSignaling) 1:1000, Cyclin D1 (92G2-CellSignaling) 1:1000, Cyclin A2 (BF683-CellSignaling) 1:1000, Total Akt (9272S-CellSignaling) 1:1000, PhosphoAkt Ser473 (664441-Proteintech) 1:1000, GAPDH (PA1-9046-Invitrogen) 1:1000. Secondary anti-rabbit HRP (111-035-144-Jackson ImmunoResearch) 1:10,000, secondary anti-mouse HRP (115-035-166-Jackson ImmunoResearch) 1:10,000.

**Results**

***The anti-tumor activity of Bromelain is mediated via apoptosis and autophagy***

Expression of active caspase 3/7 and annexin V was used to determine the effects of bromelain on tumor cell apoptosis (Figure 5). Untreated control organoids showed low annexin V staining and low caspase 3/7 activity, whereas in bromelain-treated organoids, many tumor cells were positive for annexin V and increased caspase 3/7 activity (Figure 5A). The mean fluorescence intensity (MFI) of caspase 3/7 activity from Figure 5A was calculated and presented as bar graphs in Figure 5B. Bromelain-treated organoids show a significant increase in MFI compared to control (p<0.04, n=3) suggesting that programmed cell death pathways are involved in bromelain mediated cytotoxic activity.

To further investigate how bromelain reduces tumor cell viability, we analyzed the expression of proteins involved in cell cycle regulation, cell survival, apoptotic, and autophagy pathways (Figure 5). Appendiceal cancer organoids from three patients were treated with bromelain for 48 hours at 37°C, and cellular proteins were extracted for Western blot analysis. Expression of cell cycle regulatory cyclins A2, D1, E1, and H were decreased upon bromelain treatment (Figure 5C and 5F). Similarly, the expression of anti-apoptotic Bcl-2, Bcl-xL was reduced along with the phosphorylation of serine 473 of the pro-survival protein Akt (Figure 5D and 5H).

Since autophagy may also contribute to bromelain’s induction of cell death, we analyzed the expression of autophagosome marker protein LC3-A/B I and II, and autophagy-related proteins Becline 1, Atg5, Atg7, and Atg12 (Figure 5E and 5G). The expression of LC3-A/B was increased whereas the expression of other autophagy-related proteins was decreased in organoids treated with bromelain, suggesting that bromelain causes changes in autophagy pathways and may lead to the observed increased anti-tumor activity of bromelain.

**Discussion**

The mechanisms we examined revealed that bromelain inhibited the progression of cell cycles and promoted apoptosis and autophagy in AC PTOs. Several studies have indicated that cancer progression is linked to the dysregulation of the cell cycle, which is controlled by cyclins^26^. Several cyclins have subsequently been linked to cell-cycle deregulation in tumors, particularly overexpression of cyclin E, which controls entry into S phase and centrosome replication. Cyclins A and cyclin B are expressed in the late S phase, where cyclin A initiates DNA replication and cyclin B allows for exit into G2/M phase^26,27^. In our appendiceal bromelain-treated organoids, we find inhibition of cyclin D1 and the decreased expression of cyclin A and cyclin E, indicating a natural arrest in cell cycle progression at the beginning of the G1 phase. This finding suggests that bromelain-induced growth inhibitory effects may interfere with cell cycle progression, leading to cell cycle arrest.

We also examined genes involved in the regulation of apoptosis and autophagy. The Bcl-2 family is central in controlling this complex yet tightly regulated process^28^. Within this family, Bcl-2 and Bcl-XL, inhibit cell death by preventing mitochondrial outer membrane permeabilization (MOMP)^29^. MOMP triggers the release of pro-apoptogenic factors, activating the caspase cascade that dismantles and destroys the cell. Thus, reduced expression of Bcl-2 and Bcl-XL lead to apoptosis with parallel facilitation of autophagy through decreasing Bcl-2/Bcl-XL dependent Becline-1 binding. The results of the present study indicate that bromelain treatment reduces both Bcl-2 and Bcl-XL protein levels in our PTO model with increased involvement of both activated caspases 3/7 and annexin V. Furthermore, the expression of the active form of Akt, was inhibited in bromelain-treated appendiceal cancer organoids. Akt is one of the most frequently activated protein kinases in human cancers^30^, associated with decreased apoptosis, tumor progression and resistance to chemotherapy^31-33^. Our data suggest that an imbalance between pro- and anti-apoptotic proteins in the Bcl-2 family may cause bromelain-induced apoptosis in AC PTOs.

Several studies have found that autophagy, as a type II programmed cell death, plays an essential role in cancer by expressing autophagy-related (Atg) proteins^34^. Autophagy, as a dynamic cellular breakdown and recycling system suppresses tumor growth in early stages of tumor development while facilitates growth and promotes metastasis at later stages^35^. To further study a possible crosstalk disruption between apoptosis and autophagy pathways, we examined the expression of a number of autophagy proteins. In this context, our results show inhibition of Becline-1, Atg7 and 12; and increased expression of Atg5, with increased LNC3 I and II (Figure 5E and G).
